# Assessing the feasibility of using toenails as biomarkers for estimating inorganic arsenic exposure in Japanese adults

**DOI:** 10.1265/ehpm.24-00073

**Published:** 2024-11-02

**Authors:** Tomoko Oguri, Naohide Shinohara, Shoji F. Nakayama

**Affiliations:** 1 Research Institute of Science for Safety and Sustainability (RISS), National Institute of Advanced Industrial Science and Technology (AIST), 16-1 Onogawa, Tsukuba, Ibaraki 305-8569, Japan; 2 Health and Environmental Risk Division, National Institute for Environmental Studies, 16-2 Onogawa, Tsukuba, Ibaraki 305-8506, Japan

**Keywords:** Inorganic arsenic exposure, Toenail clippings, Dietary record method, Biomarker

## Abstract

**Background:**

Long-term exposure to inorganic arsenic (InAs) through arsenic (As)-contaminated drinking water poses serious health risks. However, epidemiological studies focusing on low-level dietary exposure to InAs are lacking. Furthermore, although toenail clippings are used as biomarkers for assessing As exposure in areas with contaminated drinking water, to date, no method has been developed for using toenails as a biomarker of long-term InAs exposure in individuals with lower exposure levels, e.g., from dietary sources including fish and seaweeds. Therefore, this study aimed to assess the feasibility of using toenails as biomarkers for estimating InAs exposure in Japanese adults.

**Methods:**

Three sets of 7-day diet records and toenail clipping samples were collected from 39 healthy adult participants at intervals of 1–6 months over 4–8 months, spanning from June 2019 to March 2020. The analysis sample sets comprised 113 sample sets obtained from 38 subjects: 56 samples from 19 males and 57 samples from 19 females. The speciation of As species in the toenail samples was performed using high-performance liquid chromatography-inductively coupled plasma mass spectrometry. The sum of the InAs and monomethylarsonic acid (MMA) or sum of As species (sum-As) concentrations in toenail samples was used as an index of InAs exposure.

**Results:**

The geometric mean (GM) concentration of InAs + MMA or sum-As in toenails was 0.180 µg As/g or 0.284 µg As/g. The estimated GM of daily dietary InAs exposure was 0.147 µg/kg/day. Log-transformed InAs + MMA or sum-As concentrations in toenails did not predict dietary InAs exposure levels from rice and hijiki consumption in both males and females. Similarly, toenail InAs + MMA or sum-As concentrations showed no correlation with dietary InAs exposure levels from rice or hijiki consumption.

**Conclusions:**

Our findings indicate that human toenail clippings are not a suitable biomarker for assessing long-term InAs exposure levels in Japanese individuals based on the observed range of InAs and its metabolite concentrations in toenails.

**Supplementary information:**

The online version contains supplementary material available at https://doi.org/10.1265/ehpm.24-00073.

## Introduction

Long-term exposure to inorganic arsenic (InAs) through contaminated drinking water has been linked to an increased risk of skin, lung, bladder, and liver cancers [1], along with other health issues, making it a global health concern. The carcinogenic risk associated with InAs exposure through diet has become a cause for concern in non-contaminated areas, prompting health risk assessments worldwide [2–6].

In non-contaminated regions, rice consumption serves as a significant source of InAs exposure [7], whereas in specific areas, hijiki seaweed consumption also contributes significantly to InAs exposure [8]. Residents in such areas are often exposed to InAs through these dietary sources, potentially increasing their risk of cancer. However, limited epidemiological studies have investigated low-level InAs exposure through dietary intake in non-contaminated areas [9, 10]. In environmental epidemiological studies, having a reliable biomarker for InAs exposure would be invaluable for assessing dose-response associations in populations.

The concentrations of urinary InAs and its metabolites are commonly used biomarkers in epidemiological studies [11]. Earlier epidemiological studies measured total-As concentrations in urine since these studies targeted total-As exposure derived from drinking water. Since the toxicity of arsenic (As) depends on its chemical form, their studies analyzed the concentrations of urinary InAs and its metabolites to gather information on As species in recent studies.

It is crucial to note that these concentrations can be strongly influenced by recent InAs exposure, as ingested InAs is readily absorbed and eliminated in urine within four days [12]. Moreover, the concentrations of InAs and its metabolites in a single urine sample reflect exposure the day before from dietary InAs sources on a daily basis; it is important to exercise caution when assessing the long-term exposure to InAs in individuals. The intraclass correlation coefficient (ICC) of urinary InAs and its metabolites as a measure of reliability is 0.15–0.25, indicating poor reproducibility [13].

Arsenic (As) has a strong affinity for sulfhydryl groups found in hair and nails. Consequently, similar to those in hair, As concentrations in nail samples are indicative of blood As levels, accumulating during their formation and believed to gather in keratin-rich tissues such as hair, fingernails, and toenails [14, 15]. Unlike urine or blood samples that reflect exposure over hours or days, these solid biological matrix samples provide insights into exposure spanning several months. Among these, toenails are considered suitable for assessing long-term As exposure, as they are less exposed to external elements such as air and water compared to fingernails and scalp hair. Additionally, toenail collection is less invasive and offers the advantage of ease of collection and storage [16, 17].

Toenail clippings are often used as biomarkers of As exposure, particularly in epidemiological studies conducted in areas with As-contaminated drinking water [18–22]. However, no method has been developed for using toenail As concentration as a biomarker of long-term InAs exposure in individuals exposed to less toxic organic As compounds from diets containing fish and seaweed. Furthermore, the relationship between dietary InAs exposure and excretion in toenails must be assessed based on the species of As, particularly in individuals who consume a variety of As species. This includes toxic InAs and less toxic organic arsenic species, which are obtained from a diet that includes fish, shellfish, and seaweed, as is common among Japanese people. Therefore, it is crucial to assess the feasibility of using toenails as biomarkers before studying the dose-response association of InAs in the subject population. To address this gap in the literature, this study aimed to assess the feasibility of using toenails as biomarkers for estimating long-term InAs exposure in Japanese individuals.

## Materials and methods

### Study participants and sample collection

The present study was conducted in Tsukuba City and the surrounding area (near the Tokyo Metropolitan area) of Japan, with data collection occurring during June and October 2019 (first time), December 2019 (second time), and January to March 2020 (third time). Participants were recruited through word-of-mouth and a local research company. Interested healthy individuals were provided with research information and consent forms, and written informed consent was obtained from all participants. A total of 39 individuals participated in the study, comprising customer service workers, office workers, healthcare workers, engineers, housewives, barbers, and non-workers. This study was approved by the Ethics Committee of the National Institute of Advanced Industrial Science and Technology (AIST; approval number: Hi-2018-0277-C).

Three sets of 7-day diet records and toenail clippings were sampled from each of the 39 healthy participants at 1–6 month intervals over 4–8 months. The study included 39 healthy participants, comprising 20 males and 19 females aged 21–76 years at the time of the study. One subject was excluded from the study as they did not consume rice and hijiki seaweed in all three sampling periods, and another participant did not provide toenail samples on one occasion. At the end of the three sampling sessions, a total of 113 sets of samples were obtained. After exclusions, the analysis sample sets comprised 113 sample sets obtained from 38 subjects: 56 samples from 19 males and 57 samples from 19 females (Table 1).

**Table 1:** Characteristics of participants who provided toenail clippings and dietary records of food consumption

**Characteristics**	**Total**	**Male**	**Female**
Gender (number)	38^a^	19^a^	19
Age (years)	44.2 ± 14.0	42.6 ± 13.4	45.9 ± 14.7
Body weight (kg)	62.7 ± 11.9	68.8 ± 10.7	56.6 ± 10.0
BMI (kg/m^2^)	22.9 ± 3.2	23.1 ± 2.7	22.7 ± 3.7

Total number of biological samples collection			
Toenail samples	113	56	57
Missing values	1	1	0

Food consumption			
Rice (g/day)	265.1 ± 141.2	327.5 ± 152.0	203.8 ± 97.4
Rice, Yes (numbers)	113	56	57
Rice, No (numbers)	0	0	0
Cooked hijiki (g/day)	3.7 ± 13.8	4.1 ± 15.5	3.4 ± 12.1
Cooked hijiki, Yes (numbers)	27	17	10
Cooked hijiki, No (numbers)	86	39	47

Values are presented as mean ± standard deviation or numbers (sample numbers).

^a^One subject who did not consume rice and cooked hijiki in all three sampling periods was excluded.

Dietary InAs exposure levels were determined using a dietary record method. Participants were provided with a kitchen scale to weigh their consumption of cooked rice (brown and polished) and cooked hijiki servings during the study period. Written instructions were provided for recording the weight of cooked rice (brown and polished) and cooked hijiki consumed over seven consecutive days, including weekdays and weekends. Participants also provided information about their age, body weight, and profession.

Participants were instructed to collect toenail clippings from all ten toenails using stainless steel clippers provided in a plastic bag with zippers on the day after the dietary record sampling, if possible, after removing any nail polish. The toenail clippings were transported to the laboratory and stored in a refrigerator. All polypropylene bottles and tubes were acid-washed before use, except for the plastic bag with a zipper, which was used without prior washing.

### Preparation of toenail samples

Toenail samples were washed following a modification of a previously described protocol [17, 23]. Initially, any visible exogenous material on the toenail samples was manually removed using Teflon tweezers. Subsequently, the samples underwent a series of sonications: first, a single sonication for 1 min in ultrapure water, followed by three sonications for 1 min each in acetone, and finally, another sonication for 1 min in ultrapure water. Following this, the samples were air-dried on cellulosic filter paper (Advantec Toyo Kaisha, Ltd., Tokyo, Japan), then dried overnight in a 60 °C oven (ETTAS On-300s, AS ONE Corporation, Osaka, Japan), and weighed. To facilitate analysis, toenail clipping samples were cut into smaller pieces using stainless steel nail clippers. All samples were stored at −20 °C until analysis.

### As speciation analysis

Toenail samples were weighed and placed in perfluoroalkoxy alkane vessels containing 1 mL of HNO_3_ (AA-100, Tama Chemical Co., Ltd., Kawasaki, Japan). These vessels were then sealed and subjected to microwave digestion at 120 °C for 30 min using a microwave digestion device (START D; Milestone General, Kawasaki, Japan) and digested. After digestion, the acid was evaporated to dryness on a hot plate at 120 °C, and the residue was redissolved in 2 g of ultrapure water. The resulting solution was filtered through a 0.45-µm filter (Mini-Sarto, Sartorius, Germany). The average sample mass of nail clippings from ten toes per sampling event per person was 25 ± 4.7 mg, which was deemed sufficient for analysis.

Standard stock solutions of As(V), dimethylarsinic acid (DMA), and arsenobetaine (AB) (1000 ng As/g) were prepared using certified reference materials (NMIJ CRM 7912-a, 7913-a, and 7901-a, National Metrology Institute of Japan, Ibaraki, Japan). Similarly, a standard solution of As(III) (1000 ng As/g) was prepared from a 100 mg/kg As(III) standard solution (Kanto Chemical Industries Ltd., Tokyo, Japan), and monomethylarsonic acid (MMA) and trimethylarsine oxide (TMAsO) (1000 ng As/g) was prepared by dissolving MMA and TMAsO (Tri Chemical Co. Ltd., Yamanashi, Japan) in water. Working mixed standard solutions (1–10 ng As/g) were prepared by diluting the stock solutions with water on the day of analysis. Ultrapure water from a Milli-Q system (Milli-Q Advantage; Merck Co., Ltd., Tokyo, Japan) was used for sample preparation.

The speciation of As in toenail samples was performed using high-performance liquid chromatography-inductively coupled plasma mass spectrometry (HPLC-ICP-MS). An HPLC system (LC-2000 Plus, JASCO, Tokyo, Japan) equipped with a CAPCELL PAK C18 MG column (250 mm × 4.6 mm I.D., particle size 5 µm, Osaka Soda Co. Ltd., Osaka, Japan) and ICP-MS Agilent 7800 (Agilent Technologies, Tokyo, Japan) was used. The chromatographic and ICP-MS conditions followed those described by Narukawa and Chiba [24]. The limit of detection (LOD) was calculated based on a signal-to-noise ratio of 3.0. For a 10 µL injection, the LOD in nail samples was determined to be 0.040 As(V), As(III), and AB, and 0.050 µg As/g for MMA and DMA, and 0.10 µg As/g for TMAsO.

The total value of InAs, MMA, and DMA value was employed as an indicator for InAs exposure in individuals residing in groundwater As-contaminated areas and those with occupational InAs exposure. However, this measure is insufficient to serve as an indicator of InAs exposure in seaweed consumers due to the significant metabolism of arsenosugar into DMA, which is then metabolized and excreted in humans. Therefore, in this study, we utilized the sum of InAs and MMA concentrations as an index for InAs exposure in toenail samples of Japanese participants, as proposed by Hata et al. [25]. Additionally, information on the sum of individual As species in toenail samples has been presented as an index for InAs.

### Analytical quality control

CRMs of hair materials were measured in each batch of ten toenail samples. CRMs were used for external analytical quality control of the InAs analysis of the nail samples. The measured values of As(V), As(III), MMA, DMA, AB, and TMAsO in National Institute for Environmental Studies (NIES) CRM No. 13, Human hair, were 0.11 ± 0.01, <0.04, <0.05, <0.05, <0.04, and <0.1 µg As/g, respectively. The concentration of InAs as the sum of As(V) and As(III) agreed with the reference value (total As 0.10 µg/g). Spike recovery in toenail samples was tested and ranged from 91% to 118% when spiked at 10 ng As/g for As species, excluding AsB. Our method detected all spiked AB as TMAsO, likely due to transformation between the two forms. The recovery rate for TMAsO was 107% when spiked to 50 ng As/g standard for AB.

Therefore, the As(V) and As(III) concentrations in the toenail samples henceforth represent the total InAs concentration [sum of As(V) and As(III)] in the toenail samples in the present study.

### Estimation of dietary InAs exposure levels through rice and hijiki

Based on a previous report indicating that rice and hijiki are the main sources of InAs exposure in Japanese individuals [8], the estimated dietary InAs exposure level was calculated as follows:

$$\begin{array}{rl} & \text{Intake}\;(\text{µg/person/day})=C_{R}\times {\mathrm{IR}}_{R}+C_{H}\times {\mathrm{IR}}_{H}\\ & \text{Intake}\;(\text{µg/kg body/day})\\ & \;=\text{Intake}\;(\text{µg/person/day})÷\mathrm{BW}, \end{array}$$

where *Intake* is the estimated dietary InAs exposure level (µg/person/day or µg/kg body/day), *C_R_* is the literature data of mean concentration in rice [26], *C_H_* is the mean concentration in cooked hijiki [26]. *IR_R_* is the estimated daily consumption of rice based on the recorded weight of cooked rice consumed over a 7-day period, *IR_H_* is the estimated daily consumption of hijiki based on the recorded weight of cooked hijiki consumed over 7 days, and *BW* is the body weight of each individual in the present study.

### Statistical analysis

Descriptive statistics, Mann–Whitney U-test, Pearson test, analysis of covariance (ANCOVA), simple regression analysis, and multiple regression analysis were performed using JMP 14.3.0 (SAS Institute Japan, Tokyo, Japan). When the concentration of As species in toenail samples was below the LOD, one-half of the LOD value was substituted for statistical analyses. The sum of individual As species (sum-As) was calculated by substituting nondetectable values with one-half the LOD value. Given that dietary InAs exposure levels and As species concentrations in the toenail samples did not follow a normal distribution, they were logarithmically transformed for statistical analyses.

Multiple regression analysis was performed using the forced-entry method. The toenail concentration of InAs + MMA or sum-As served as the independent variable, whereas the dietary InAs exposure level, derived from the combined consumption of rice and/or hijiki, was the dependent variable. Age (in years) and body mass index (BMI) were included as covariates, reflecting fundamental biological attributes.

We first conducted simple and multiple regression analyses on 113 sets of samples obtained from 38 subjects (19 males and 19 females) across three sampling sessions. Subsequently, the regression analysis of dietary InAs intake and toenail InAs metabolite concentrations, based on the average values of the three sampling sessions, was performed and is presented in the supplemental data (Table S2). Given that regression analysis assumes a normal distribution and outliers can distort representative values, data filtered for extreme outliers were used, including one InAs sample that had values more than three standard deviations above the mean [27].

## Results

### Participants and dietary records

Table 1 presents the characteristics of the study participants. The 113 sets of samples comprised 56 samples of males (aged 42.6 ± 13.4 years) and 57 samples of females (aged 45.9 ± 14.7 years). The mean body weights of the male and female participants were 68.5 ± 10.1 and 57.0 ± 9.9 kg, respectively, with corresponding BMIs of 23.0 ± 2.7 and 22.8 ± 3.6 kg/m^2^.

Dietary records for 2394 meals (38 participants × 3 times × 7 days × 3 meals per day) were completed by the participants. Rice and cooked hijiki were served in 1186 meals (49.5%) and 58 meals (2.4%), respectively, with consumption frequencies of 44.6 and 2.2 meals/month for rice and hijiki, respectively.

### Dietary exposure levels of InAs and concentrations of InAs and its metabolites in toenails

**Fig. 1:**
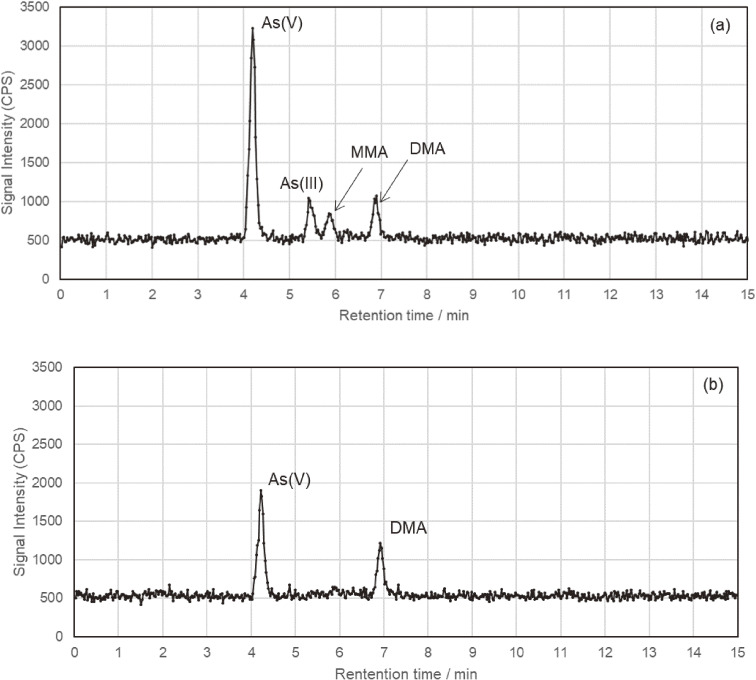
Typical LC-ICPMS chromatograms of nitric acid digestion at 120 °C for 30 minutes, extracted from the toenails of Japanese participants. AB was not detected in these chromatograms. Chromatograms (a) and (b) show results from different subjects.

Table 2 presents the dietary InAs exposure levels and the concentrations of InAs and its metabolites in toenail samples. The dietary InAs exposure level was calculated by multiplying the reported concentration values in rice and hijiki [26] by the daily consumption amounts of these foods obtained in this study. InAs was detected in 95% of toenail samples, whereas the detection rates for MMA (3%), DMA (3%), AB (2%), and TMAsO (0%) were relatively low. Figure 1 shows the typical chromatograms of toenail samples from various subjects.

**Table 2:** Dietary InAs exposure levels and the concentrations of InAs and its metabolites in toenail samples

	**Unit**	**Male** **GM (GSD)**	**Range**	**%^c^**	**Female** **GM (GSD)**	**Range**	**%^c^**	**Total** **GM (GSD)**	**Range**	**%^c^**	***p* ^a^**
N		56			57			113			
Diet (rice + hijiki)											
InAs	µg/kg/day	0.173 (1.85)	0.0624–1.64		0.125 (2.28)	0.0170–1.36		0.147 (2.10)	0.0170–1.64		<0.05
Rice											
InAs	µg/kg/day	0.138 (1.61)	0.0524–0.311		0.101 (1.82)	0.986–13.2		0.118 (1.76)	0.0170–0.311		<0.05
Hijiki											
InAs	µg/kg/day	0.0888 (3.31)	0.0215–1.48		0.183 (3.65)	0.0261–1.28		0.116 (3.66)	0.0215–1.48		ns^b^

Toenail											
InAs + MMA	µg/g	0.176 (1.47)	0.065–0.477	64	0.184 (1.85)	<0.13–5.70	63	0.180 (1.67)	<0.13–5.70	63	ns^b^
InAs	µg/g	0.146 (1.58)	<0.080–0.392	53	0.156 (1.96)	<0.080–5.67	54	0.151 (1.78)	<0.080–5.67	53	ns^b^
MMA	µg/g	<0.050	<0.050–0.0849	(9)	<0.050	<0.050	(9)	<0.050	<0.050–0.0849	(9)	ns^b^
DMA	µg/g	<0.050	<0.050–0.059	(9)	<0.050	<0.050–0.079	(9)	<0.050	<0.050–0.0788	(9)	ns^b^
AB	µg/g	<0.040	<0.040–0.0504	(7)	<0.040	<0.040	(7)	<0.040	<0.040–0.0504	(7)	ns^b^
TMAsO	µg/g	<0.10	<0.10	(18)	<0.10	<0.10	(17)	<0.10	<0.10	(18)	
Sum-As	µg/g	0.277 (1.28)	<0.32–0.571		0.290 (1.62)	<0.32–5.79		0.284 (1.47)	<0.32–5.79		ns^b^

a Mann–Whitney U-test for gender-related difference

b Not significant.

c Percentage to sum-As

In this study, the geometric means of dietary InAs exposure, toenail InAs + MMA, and toenail sum-As concentration for all 113 sets of samples were 0.147 µg/kg/day, 0.180 µg/g, and 0.284 µg/g, with ranges of 0.017 to 1.64 µg/kg/day, <0.13 to 5.70 µg/g, and <0.32 to 5.79 µg/g, respectively. Detected As species in toenails were predominantly InAs species, with low detection rates for organic As species (Table 2).

No significant differences in toenail As species concentrations were observed between sexes (*p* > 0.05, Mann–Whitney’s U-test). However, a significant sex difference (male > female) was noted for dietary InAs exposure levels (Table 2). Table S1 shows the information provided regarding the differences between the three sampling sessions.

### Correlation between toenail levels of InAs and its metabolites and daily dietary InAs exposure level

Table 3 presents the toenail InAs + MMA or sum-As concentrations and dietary InAs exposure from the sum of rice and/or hijiki in the multiple regression models on 113 sets of samples obtained from 38 subjects during the three sampling sessions. Regression analysis was conducted separately for male and female samples due to gender-based differences in the regression equations identified by ANCOVA (data not shown).

**Table 3:** Dietary InAs exposure levels as predictors of the concentrations of toenail InAs and its metabolites

**Analyte**	**Variable**	**β^a)^ [95%CI^b)^]**		**R^2 c)^**	***p-value***
**Male**					
Model 1					
Dietary InAs exposure level	Nail InAs + MMA	0.131	[−0.220, 0.636]	0.017	0.335
Rice InAs exposure level		0.363	[0.133, 0.761]	0.132	<0.05
Hijiki InAs exposure level		−0.061	[−2.326, 1.861]	0.004	0.816
Dietary InAs exposure	Nail sum-As	0.099	[−0.424, 0.917]	0.010	0.465
Rice InAs exposure level		0.332	[0.144, 1.135]	0.111	0.012
Hijiki InAs exposure level		−0.084	[−3.867, 2.834]	0.007	0.747
Model 2					
Dietary InAs exposure level	Nail InAs + MMA	0.148	[−0.204, 0.674]	0.038	0.289
Rice InAs exposure level		0.363	[0.123, 0.772]	0.135	<0.01
Hijiki InAs exposure level		−0.031	[−2.312, 2.076]	0.095	0.909
Dietary InAs exposure	Nail sum-As	0.111	[−0.413, 0.964]	0.029	0.426
Rice InAs exposure level		0.334	[0.131, 1.156]	0.115	<0.05
Hijiki InAs exposure level		−0.079	[−3.992, 3.024]	0.100	0.770

**Female**					
Model 1					
Dietary InAs exposure level	Nail InAs + MMA	0.085	[−0.376, 0.723]	0.007	0.530
Rice InAs exposure level		0.245	[−0.027, 0.754]	0.060	0.068
Hijiki InAs exposure level		−0.342	[−3.306, 1.264]	0.117	0.333
Dietary InAs exposure	Nail sum-As	0.074	[−0.598, 1.049]	0.006	0.585
Rice InAs exposure level		0.239	[−0.056, 1.116]	0.057	0.076
Hijiki InAs exposure level		−0.363	[−5.117, 1.807]	0.132	0.303
Model 2					
Dietary InAs exposure level	Nail InAs + MMA	0.085	[−0.391, 0.738]	0.008	0.541
Rice InAs exposure level		0.249	[−0.025, 0.763]	0.095	0.066
Hijiki InAs exposure level		0.052	[−2.847, 3.162]	0.466	0.902
Dietary InAs exposure level	Nail sum-As	0.074	[−0.620, 1.068]	0.006	0.597
Rice InAs exposure level		0.242	[−0.053, 1.125]	0.092	0.074
Hijiki InAs exposure level		0.047	[−4.571, 5.003]	0.466	0.916

Model 1: unadjusted.

Model 2: adjusted for age and BMI

a) β, standardized regression coefficient; b) CI, confidence interval; c) R^2^, Coefficient of determination

In models adjusted for age and BMI, no evidence of an association was found between toenail InAs + MMA or sum-As concentrations and dietary InAs exposure from the sum of rice and hijiki in both males and females. However, a significant association was observed between male toenail InAs + MMA or sum-As concentrations and iAs intake from rice consumption alone, although the reliability of the regression equation was low (*β* = 0.36, R^2^ = 0.13; *β* = 0.33, R^2^ = 0.11). Conversely, toenail InAs + MMA concentrations showed no correlation with dietary InAs exposure levels from consuming only rice or only hijiki (Table 3). Table S2 presents regression analysis results based on the average of three sampling sessions per person, showing similar trends to those in Table 3.

Table 4 presents a comparison of the correlation coefficients between InAs and its metabolite concentrations in toenails and dietary InAs exposure levels in the present study with values reported in previous studies.

**Table 4:** Comparison of the findings obtained in the present study with those reported in previous studies.

**Location**	**Dietary As exposure levels (µg/kg/day)**	**Nail As (µg/g)**	**Correlation coefficient (*r*)**		**Reference**
	**Mean (range)**	**Mean (range)**			
Japan	0.202 (0.0170–1.64) (diet)	0.238 (<0.13–5.70) (InAs+MMA) 0.335 (<0.32–5.79) (sum-As)	0.131 (M^b^), 0.018 (F^c^) (InAs+MMA) 0.099 (M^b^), 0.073 (F^c^) (sum-As)	InAs (diet, rice) InAs + MMA or sum-As (toenail)	The present study
	0.135 (0.0170–0.311) (rice)		0.363 (M^b^), 0.146 (F^c^) (InAs+MMA) 0.332 (M^b^), 0.239 (F^c^) (sum-As)		
Inner Mongolia	0.370 (0.260–0.910)	0.329 (0.084–1.29)	0.47	Total As (food + water, nail)	[37]
Cambodia	5.31 (1.16–19.5)	0.830 (0.099–2.38)^a^	0.555	InAs (diet), Total As (nail)	[38]
Pakistan	3.22 (0.02–236) (water)	1.76 (0.557–22.0)	0.766^b^	Total As (water, food diet), -InAs (nail)	[39]
	0.589 (0.275–2.02) (stable food)				
China	2.6 (0.2–14.1)	7.8	0.412	Total As (diet, nail),	[40]
Bangladesh	2.2 (<1–30.2) (Drinking water)	1.26 (0.20–5.53)	0.830^b^	Total As (water, food, nail)	[36]
	1.2 (<1–7.1) (Food)				

a) Fingernail; b) male; c) female.

## Discussion

A strength of the present study is that it includes Japanese individuals residing in areas with non-contaminated groundwater. In this study, the concentrations of InAs and its metabolite in the toenails as well as dietary InAs exposure levels were examined. While the urine concentration of InAs and its metabolites has been recognized as a suitable biomarker in environmental epidemiological studies in areas with As-contaminated groundwater, it has limitations in terms of reflecting exposure over a short time period. InAs is easily metabolized and eliminated in the urine within four days [12]. However, toenails have a slow growth rate, and nail clipping samples can reflect the growth that occurred 10 months ago [28]. Thus, in the present study, we tested the hypothesis that a biomarker single toenail clipping indicates long-term internal exposure to InAs in Japanese individuals. To the best of our knowledge, this is the first study to evaluate toenails as indicators of InAs exposure in fish, shellfish, and seaweed consumers, such as the Japanese.

The geometric mean (GM) of the dietary InAs exposure level in the present study was 0.15 µg/kg/day, which aligns with recently reported InAs exposure levels among Japanese individuals, within the compass of their variability. Hayashi et al. [29] measured dietary InAs exposure levels in a duplicate diet study from 319 households, reporting a GM of 0.270 µg/kg/day with a range of <0.05 to 1.5 µg/kg/day. Similarly, Yoshinaga and Narukawa [30] conducted a duplicated diet study, reporting a GM dietary InAs exposure level of 150 households in Japan as 0.349 µg/kg/day. They also found a significant positive correlation between dietary InAs exposure levels and urinary InAs + MMA excretion. Oguri et al. [31] found a GM of dietary InAs exposure level among 39 participants to be 0.087 µg/kg/day (range: <0.028 to 0.50 µg/kg/day), and the observed range of InAs exposure from the diet showed a significant association with urinary iAs + MMA concentrations. These comparisons with previous studies in Japanese reveal moderate variations in dietary InAs exposure levels both between and within study participant groups, and there was a correlation with the concentration of urinary InAs metabolites [32].

In Japan, Tabata et al. (2006) [33] conducted a study on toenails in Japan and reported that the mean levels of total As in toenail samples of 159 participants living on Amami-Oshima Island in Japan were 0.41 µg/g. A comparable study in areas without As contamination reported median total-As concentration in toenails to be 0.049 to 0.086 µg As/g among individuals in the US [34] and Canada [35]. In areas with moderate As contamination of groundwater in Bangladesh, a mean value of 1.26 µg As in nail samples was reported, with approximately one-third exceeding the drinking water standard of 50 µg/L [36]. Our results of sum-As concentrations coincide with previous values reported by Tabata et al. [33].

One of the few studies examining As speciation in nails from the As-contaminated groundwater area of West Bengal, India—conducted by Mandel et al. [22]—found concentrations of As(iii) at 1.81 µg/g, As(V) at 0.59 µg/g, MMA(V) at 0.16 µg/g, DMA(III) at 0.26 µg/g, and DMA(V) at 0.08 µg/g in fingernail samples. Conversely, most As species in toenail samples in the present study were detected in the InAs forms. The InAs concentrations reported in this paper were more than ten times higher than those in the present study, suggesting that InAs exposure levels were expected to be high and that detectable levels of organic As species, as InAs metabolites, were likely present in the nails.

For Japanese individuals, dietary InAs exposure levels primarily stem from rice and hijiki [8]. Therefore, in the present study, we initially estimated the long-term average InAs exposure level based on dietary records of rice and hijiki consumption over a 7-day period. Additionally, a previous study suggested that mean regular daily dietary InAs exposure levels could be collected and measured in urine over a 4-day period based on repeatability calculations with repeated sampling [14]. The diet record period in the present study was set at 7 days.

The average dietary InAs exposure levels were then examined for relationships with the measured concentrations of InAs and its metabolites in toenail clippings collected from each individual on the day following the conclusion of the dietary records. Results from multiple linear regression indicated that the levels of InAs and its metabolites in toenails were not predictors of dietary InAs exposure levels resulting from the sum of rice and/or hijiki consumption in both males and females (Table 3). These results indicate that assessing InAs exposure using a single toenail clipping biomarker within the range of concentrations of InAs and its metabolites observed in Japanese toenails may prove challenging.

Our results demonstrated lower values of correlation coefficients (*r*) (i.e., ≤0.3), indicating no association between the concentrations of InAs and its metabolites in toenails and dietary As exposure levels compared to previous studies (Table 4). Previous studies have provided limited insights into the relationship between nail concentration and dietary As exposure (Table 4), and these studies were conducted in areas contaminated with As groundwater. These studies revealed correlation coefficients ranging from 0.41 to 0.83, with moderate correlations (i.e., ranging from >0.4 to <0.7) observed in studies from Inner Mongolia, Cambodia, Pakistan, and China with known drinking water As contamination (mean concentration of As 50 to 170 µg/L) [37–40]. A strong correlation (i.e., >0.7) was found in a study from Bangladesh [36]. Studies conducted in areas with groundwater contaminated with As, such as Bangladesh and Inner Mongolia, Cambodia, Pakistan, and China, have identified drinking water as the main source of As exposure (Table 4).

However, in the Japanese population, exposure to InAs from rice and hijiki seaweeds appears to be the main source of InAs exposure [8]. This exposure is primarily derived from rice, which is a staple food, and hijiki, which is occasionally consumed as a side dish. Hijiki seaweed contains high levels of InAs, as is well known [41]. The primary difference between these exposure sources lies in the patterns of exposure. As exposure from drinking water shows a consistent pattern, whereas InAs exposure from hijiki seaweed results in intermittent exposure patterns. A total of 38 participants included in our study reported consuming 44.6 servings of rice and 2.2 servings of cooked hijiki per month. Another dietary record survey based on 1500 Japanese individuals reported a consumption of 2.5 servings of cooked hijiki per month [42]. These results suggest that the concentration of InAs and its metabolite in toenail may not represent long-term average exposure levels in populations with intermittent exposure patterns to InAs.

While daily dietary InAs exposure levels were shown to be associated with urinary InAs and metabolite concentrations indicating recent exposure in the Japanese individuals [30, 31], the concentrations of InAs and its metabolites in toenails indicating long-time exposure showed limited explanatory power as indices of long-term exposure to dietary InAs in the present study. Moreover, the body distribution and excretion to the nail, which serves as a secondary excretion pathway of As (the first excretion route of InAs is urine), were less pronounced, potentially complicating the determination of the relationship with dietary InAs exposure.

Therefore, our findings do not provide evidence that the concentration of InAs and its metabolite in toenails may not reflect daily dietary InAs exposure levels in areas with moderate to high groundwater contamination levels.

A significant gender-based difference was observed in the regression equations between the concentrations of toenail InAs and its metabolites and dietary InAs exposure levels, consistent with findings from a previous study [43]. Lindberg et al. [43] proposed that females exhibit a more efficient metabolism of InAs compared to males owing to the influence of sex hormones, and our results support the hypothesis that there is a distinct gender-related difference in the pattern of As excretion in toenails.

However, the present study has several limitations. First, the representativeness of the results in terms of the general Japanese adult population may be lacking because the number of subjects was moderate. The present survey was based on an *ad hoc* type survey to assess the feasibility of using toenails as InAs biomarkers in Japanese individuals. However, the accumulation of similar studies might provide a more comprehensive understanding of the availability of InAs biomarkers in non-As-contaminated areas.

Second, there were differences in the timing between the dietary InAs exposure level survey and the growth period of sampled toenails. Because the big toenail had an average length of 20 mm and a growth rate of 2.1 mm/month on average, a single nail clipping sample reflects past exposure over 10 months [28]. In this study, the mean dietary InAs exposure level was estimated through a continuous 7-day diet record survey. Based on a report by Oguri et al. [13], the mean dietary InAs exposure level over several months can be collected and measured in urine over a 4-day period. However, the limited number of participants in this study may not necessarily reflect the long-term exposure level of InAs for individuals. Third, genomic differences in the InAs methylation capacity were not considered in the present study. Further studies should involve a larger number of participants and nail samples. For instance, nail sampling over a longer period (10 months) can provide insight into the variability of ongoing InAs exposure in each segment (toenail clippings).

Toenails have been used as a biomarker for assessing InAs exposure in areas with As-contaminated groundwater. We found that the concentration of InAs and its metabolites in toenails was little associated with dietary InAs exposure in the Japanese subjects. Although this study was conducted in an *ad hoc* manner and may lack generalizability, we can conclude that within the range of InAs levels observed in the present study, the concentrations of InAs and its metabolites in a single toenail clipping may not be suitable biomarkers of long-term exposure levels in adult Japanese subjects.

## Abbreviations

ANCOVA: analysis of covariance
As: arsenic
AB: arsenobetaine
BMI: body mass index
CRMs: certified reference materials
DMA: dimethylarsinic acid
GM: geometric mean
GSD: geometric standard deviation
HPLC-ICP-MS: high-performance liquid chromatography-inductively coupled plasma mass spectrometry
HNO_3_: nitric acid
ICC: Intraclass correlation coefficient
InAs: inorganic arsenic
LOD: limit of detection
MMA: monomethylarsonic acid
SD: standard deviation
TMAsO: trimethylarsine oxide

## Supplementary information

**Additional file 1:** **Table S1** Dietary InAs exposure levels and concentrations of InAs and its metabolites in toenail samples from each sampling session. **Table S2** Analysis results based on representative values from three sampling sessions, using dietary InAs exposure levels as predictors of concentrations of toenail InAs and its metabolites.

## Declarations

### Availability of data and materials

Data used in the present study are not open to the public.
