# Supplementary material for: Assessing the feasibility of using toenails as biomarkers for estimating inorganic arsenic exposure in Japanese adults
Source: Environ Health Prev Med. 2024 Nov 2;29:59. doi: 10.1265/ehpm.24-00073 (PMC11551441; doi:10.1265/ehpm.24-00073)
Supplement: Supplementary file 1 — Additional file 1: Table S1 Dietary InAs exposure levels and concentrations of InAs and its metabolites in toenail samples from each sampling session. Table S2 Analysis results based on representative values from three sampling sessions, using dietary InAs exposure levels as predictors of concentrations of toenail InAs and its metabolites. [file ehpm-29-059-s001.docx]

**Supplementary data**

Table S1 Dietary InAs exposure levels and concentrations of InAs and its metabolites in toenail samples from each sampling session

| Sampling session |  | 1 | | | | 2 | | | | 3 | | | | Average of three sampling session | | | |
| --- | --- | --- | --- | --- | --- | --- | --- | --- | --- | --- | --- | --- | --- | --- | --- | --- | --- |
|  | Unit | Male | Female | Total | *p^a^* | Male | Female | Total | *p^a^* | Male | Female | Total | *p^a^* | Male | Female | Total | *p^a^* |
|  |  | GM (GSD) | GM (GSD) | GM (GSD) |  | GM (GSD) | GM (GSD) | GM (GSD) |  | GM (GSD) | GM (GSD) | GM (GSD) |  | GM (GSD) | GM (GSD) | GM (GSD) |  |
| N |  | 19 | 19 | 38 |  | 18 | 19 | 37 |  | 19 | 19 | 38 |  | 19 | 19 | 38 |  |
| Diet (rice + hijiki) InAs | μg/kg/day | 0.198 (2.14) | 0.138 (2.13) | 0.166 (2.16) | ns^b^ | 0.164 (1.58) | 0.121 (2.37) | 0.141 (2.02) | ns^b^ | 0.160 (1.80) | 0.114 (2.42) | 0.135 (2.13) | ns^b^ | 0.190 (1.67) | 0.134 (2.18) | 0.159 (1.96) | ns^b^ |
| Rice InAs | μg/kg/day | 0.133 (1.61) | 0.104 (1.74) | 0.118 (1.69) | ns^b^ | 0.135 (1.60) | 0.101 (1.87) | 0.117 (1.76) | ns^b^ | 0.147 (1.67) | 0.0959 (1.92) | 0.119 (1.85) | <0.05 | 0.143 (1.53) | 0.103 (1.75) | 0.121 (1.68) | ns^b^ |
| Hijiki InAs | μg/kg/day | 0.107 (4.81) | 0.177 (2.12) | 0.130 (3.67) | ns^b^ | 0.0562 (2.46) | 0.146 (7.01) | 0.0750 (3.54) | ns^b^ | 0.205 (1.19) | 0.272 (8.88) | 0.237 (3.58) | ns^b^ | 0.0911 (3.58) | 0.165 (3.73) | 0.114 (3.60) | ns^b^ |
| Toenail  InAs + MMA | μg/g | 0.148 (1.33) | 0.144 (1.50) | 0.166 (1.93) | ns^b^ | 0.236 (1.39) | 0.252 (1.41) | 0.244 (1.40) | ns^b^ | 0.158 (1.47) | 0.133 (1.31) | 0.145 (1.40) | ns^b^ | 0.183 (1.31) | 0.183 (1.28) | 0.183 (1.29) | ns^b^ |
| Toenail InAs | μg/g | 0.117 (1.42) | 0.115 (1.73) | 0.136 (2.04) | ns^b^ | 0.206 (1.42) | 0.226 (1.46) | 0.216 (1.44) | ns^b^ | 0.130 (1.60) | 0.107 (1.39) | 0.118 (1.51) | ns^b^ | 0.155 (1.37) | 0.157 (1.33) | 0.156 (1.35) | ns^b^ |
| Toenail MMA | μg/g | <0.05 | <0.05 | <0.05 |  | <0.05 | <0.05 | <0.05 |  | <0.05 | <0.05 | <0.05 |  | <0.05 | <0.05 | <0.05 |  |
| Toenail DMA | μg/g | <0.05 | <0.05 | <0.05 |  | <0.05 | <0.05 | <0.05 |  | <0.05 | <0.05 | <0.05 |  | <0.05 | <0.05 | <0.05 |  |
| Toenail AB | μg/g | <0.04 | <0.04 | <0.04 |  | <0.04 | <0.04 | <0.04 |  | <0.04 | <0.04 | <0.04 |  | <0.04 | <0.04 | <0.04 |  |
| Toenail sum-As | μg/g | 0.198 (1.25) | 0.193 (1.34) | 0.195 (1.30) | ns^b^ | 0.283 (1.32) | 0.299 (1.35) | 0.291 (1.33) | ns^b^ | 0.207 (1.33) | 0.182 (1.32) | 0.194 (1.29) | ns^b^ | 0.232 (1.24) | 0.230 (1.22) | 0.231 (1.23) | ns^b^ |

a Mann-Whitney U-test for gender-related difference

b Not significant.

Table S2 Analysis results based on representative values from three sampling sessions, using dietary InAs exposure levels as predictors of concentrations of toenail InAs and its metabolites

| Analyte | Variable | β^a)^ [95%CI^b)^] | | R^2^ ^c)^ | *p value* |
| --- | --- | --- | --- | --- | --- |
| **Male** |  |  |  |  |  |
| Model 1 |  |  |  |  |  |
| Dietary InAs exposure | Nail InAs + MMA | 0.280 | [-0.393, 1.440] | 0.078 | 0.244 |
| Rice InAs exposure level |  | 0.650 | [0.408, 1.619] | 0.423 | <0.01 |
| Hijiki InAs exposure level |  | 0.331 | [-2.734, 6.868] | 0.109 | 0.349 |
| Dietary InAs exposure | Nail sum-As | 0.240 | [-0.731, 2.102] | 0.057 | 0.321 |
| Rice InAs exposure level |  | 0.616 | [0.509, 2.427] | 0.380 | <0.01 |
| Hijiki InAs exposure level |  | 0.291 | [-4.768, 10.46] | 0.085 | 0.413 |
| Model 2 |  |  |  |  |  |
| Dietary InAs exposure | Nail InAs+MMA | 0.312 | [-0.410, 1.577] | 0.106 | 0.229 |
| Rice InAs exposure level |  | 0.679 | [0.406, 1.709] | 0.448 | <0.01 |
| Hijiki InAs exposure level |  | 0.423 | [-4.135, 9.424] | 0.185 | 0.376 |
| Dietary InAs exposure | Nail sum-As | 0.270 | [-0.773, 2.317] | 0.082 | 0.303 |
| Rice InAs exposure level |  | 0.652 | [0.519, 2.586] | 0.411 | <0.01 |
| Hijiki InAs exposure level |  | 0.368 | [-7.949, 15.14] | 0.144 | 0.474 |
| **Female** |  |  |  |  |  |
| Model 1 |  |  |  |  |  |
| Dietary InAs exposure | Nail InAs+MMA | 0.002 | [-1.609, 1.626] | 0.000 | 0.991 |
| Rice InAs exposure level |  | 0.354 | [-0.284, 1.898] | 0.125 | 0.137 |
| Hijiki InAs exposure level |  | -0.373 | [-8.677, 4.774] | 0.139 | 0.465 |
| Dietary InAs exposure | Nail sum-As | -0.103 | [-0.974, 0.645] | 0.010 | 0.674 |
| Rice InAs exposure level |  | 0.090 | [-0.481, 0.688] | 0.008 | 0.713 |
| Hijiki InAs exposure level |  | -0.347 | [-12.78, 7.391] | 0.120 | 0.499 |
| Model 2 |  |  |  |  |  |
| Dietary InAs exposure | Nail InAs+MMA | 0.006 | [-1.789, 1.830] | 0.000 | 0.980 |
| Rice InAs exposure level |  | 0.389 | [-0.293, 2.068] | 0.181 | 0.130 |
| Hijiki InAs exposure level |  | 0.233 | [-14.46, 16.90] | 0.555 | 0.770 |
| Dietary InAs exposure | Nail sum-As | -0.102 | [-1.042, 0.715] | 0.010 | 0.697 |
| Rice InAs exposure level |  | 0.085 | [-0.524, 0.719] | 0.048 | 0.742 |
| Hijiki InAs exposure level |  | -0.250 | [-20.82, 24.71] | 0.559 | 0.748 |

Table S2 show analysis results 38 subjects based on representative values of three sampling results.

Model 1: unadjusted.

Model 2: adjusted for age and BMI.

a) β, standardized regression coefficient; b) CI, confidence interval; c) R2, Coefficient of determination
